# Occupational health surveillance of compressed air workers: a scoping review

**DOI:** 10.1093/occmed/kqag011

**Published:** 2026-05-23

**Authors:** T P Van Rees Vellinga, C T Hulshof, P J A M Van Ooij, J G Daams, F J H Van Dijk, R A Van Hulst

**Affiliations:** Department of Public and Occupational Health, Amsterdam University Medical Center, Amsterdam, the Netherlands; Medical Centre Hyperbaric Oxygen Therapy (MCHZ), Goes, the Netherlands; Department of Public and Occupational Health, Amsterdam University Medical Center, Amsterdam, the Netherlands; Diving Medical Centre, Royal Netherlands Navy, PO Box 10,000 1781 ZX Den Helder, the Netherlands; Department of Respiratory Medicine, Amsterdam University Medical Centre (AUMC), University of Amsterdam, Amsterdam, the Netherlands; Department of Medical Library Research Support Amsterdam UMC, Amsterdam University Medical Center, Amsterdam, the Netherlands; Department of Public and Occupational Health, Amsterdam University Medical Center, Amsterdam, the Netherlands; Learning and Developing Occupational Health (LDOH) Foundation, Leiden, the Netherlands; Diving Medical Centre, Royal Netherlands Navy, PO Box 10,000 1781 ZX Den Helder, the Netherlands; Department Anesthesiology/Hyperbaric Medicine, Amsterdam University Medical Center, Amsterdam, the Netherlands

## Abstract

**Background:**

Compressed air is increasingly used in the underground construction of caissons and tunnels. New risks have been identified, suggesting the need to develop counter-measures.

**Aims:**

This scoping review aimed to provide insight into the health risks, prevention and surveillance measures, and adverse health effects related to compressed air work during tunnel construction.

**Methods:**

Health risks were assessed based on the Joanna Briggs Institute Reviewers’ Manual, the recommendations of Pollock and the PRISMA Scoping Reviews Checklist. The scoping review mapped and analysed studies of compressed air work over the past 50 years. Only 45 studies were deemed eligible for inclusion in this review.

**Results:**

In the early days of mechanized tunnel construction, the incidence of decompression sickness was high, affecting up to 2032 individuals, with an incidence rate of the bends as high as 0.5%. Improvements in decompression tables and the use of mixed gases have reduced these rates to below 0.1%. New construction techniques have introduced new health risks, including silicosis. Because of traffic demands, especially in urban areas, newer tunnels have been built for operation at greater depths (up to 106 metres sea water).

**Conclusions:**

Relatively few studies in the past 50 years have analysed the effects of compressed air use for tunnel construction. Adverse health effects have decreased substantially over time, but several new health risks have been described. The registration of key information on health and safety risks and adverse health effects, in a highly accessible international repository is recommended.

Key learning points

**What is already known about this subject:** 

- In the 1970s, the incidence and prevalence of decompression sickness and dysbaric osteonecrosis were high.
- Occupational health surveillance programmes in compressed air work (CAW) primarily focused on pressure-related health effects.
- Despite the existence of an International Tunnelling Association, few scientific studies have analysed the effects of CAW worldwide.

**What this study adds:** 

- The quality of studies is generally moderate or low, with missing information on the study population, the numbers of participants, the effects of physically demanding work or aspects of work organization such as outsourcing.
- The number of tunnels being constructed at greater depth has increased.
- The studies identified are publications on risks, health effects and the application of various health surveillance measures.

**What impact this may have on practice or policy:** 

- These results suggest the need to develop an international health and safety registration system, as an extension of the International Tunnelling and Underground Space Association guidelines.
- The health surveillance documents should be deposited in a highly accessible international repository.
- International collaboration with an independent expertise centre in CAW can improve scientific insight on the health and safety of workers.

## Introduction

In many countries, underground stations and bridge foundations are constructed using compressed air caissons. Caissons are bottomless boxes sealed at the top and filled with compressed air to keep water out at depth. At present, many tunnels are built using tunnel-boring machines (TBMs), which are long tubes divided into two sections by a watertight bulkhead. The front section contains the drilling wheel, whereas the rear section houses the electric motors and machinery required to install tunnel segments. The front part is pressurized when maintenance work is necessary.

Compressed air work (CAW) is often performed in confined spaces, and workers are exposed to elevated gas pressures, potentially toxic gases, and occasionally other chemical substances, such as silica dust and diesel exhaust. Decompression after work under pressure can lead to bubble formation in the blood, increasing the risk of decompression sickness (DCS) and other serious health issues, such as delayed dysbaric osteonecrosis (DON) [1]. This review focuses on the occupational health effects and surveillance of workers involved in CAW in caissons or during tunnel construction.

This review presents current knowledge of occupational diseases in compressed air workers, including the prevention and treatment of these conditions. The term ‘occupational disease’ has been defined as ‘any disease contracted as a result of exposure to risk factors arising from work activity’ [2]. The most common occupational diseases among compressed air workers are DCS and DON [3–5]. CAW can be necessary during tunnel construction or caisson work to prevent water from flooding the work site. When exposed to compressed air, body tissues absorb large amounts of nitrogen. As workers return to the surface, nitrogen gas flows out of tissues into the circulation, and, under special circumstances, bubbles form, potentially leading to injuries such as acute DCS [6]. Symptoms can range from dysfunction of the central nervous system and spinal cord to Meniere’s disease, together with cardiopulmonary involvement and joint pain. The incidences of these occupational diseases have fallen sharply in the past 40 years, mainly due to the introduction of oxygen during decompression, which reduces uptake of inert gases and allows these gases to exit body tissues. The development of microbubbles, however, is believed to be an ongoing process [7]. In 1911, Bornstein and Plate noted bone changes in workers exposed to compressed air as delayed effects of long-term exposure [1]. The onset of DON typically manifests after several months to years of exposure. The growing number of caisson and tunnel projects has led to significant increases in the incidence of rate of DON [5]. Bone lesions correlate with the depth at which work is performed and become evident at depths exceeding 30 m, usually in the heads of the humerus and femur, and the shafts of the femur and tibia [1,5,8]. In 1977, Jones advocated comprehensive medical supervision to prevent osteonecrosis, including pre-employment examinations, shorter working hours, use of ‘Washington State decompression tables’ and employment of oxygen during decompression [4]. After the introduction of oxygen breathing during decompression, the incidence of DCS and DON reduced.

Although most studies of the effects of CAW have focused on DCS and DON, other health effects may be significant. Inhaled gases may display toxic properties as pressure increases. For example, nitrogen narcosis is a risk when inhaling compressed air with high partial nitrogen pressures. Increased nitrogen concentration in the bloodstream decreases subject ability to concentrate and make judgements, but these effects can be reversed by decreasing pressure. Tunnel construction involves working in an enclosed space. With the use of shotcrete silica particles can become airborne. Diesel engines generate harmful diesel exhaust gases [9]. Therefore, the use of silica and diesel exhaust can be a significant risk. From a preventive point of view, these exposures should be prevented where possible and monitored.

Occupational diving physicians, typically operating within a multidisciplinary team of occupational health and safety professionals, can prevent most occupational diseases associated with CAW. A risk inventory is a necessary base of appropriate measures, including health surveillance [10]. Comprehensive occupational health surveillance includes estimating the scope of the problem, monitoring trends in its incidence and identifying discernible opportunities for prevention [11]. Health surveillance programmes ideally incorporate pre-employment medical examinations, health monitoring, medical record-keeping and feedback regarding the preventive measures undertaken. Health-related monitoring complements exposure assessments for evaluating health risks [10]. Each time prior to starting work in saturation environments, compressed air workers are examined for skin and ear infections, as well as for impaired lung and heart function. In accordance with the recommendations of the Health and Safety Executive in the UK, there is a distinction between health records and medical records. Health records organized by employers contain information about exposure to specific chemical substances [12]. Medical records are usually a subset of health records and may contain confidential medical information stemming from the occupational health physicians. Analysing and publishing health records is complicated by this dual ownership of the information of the health record. At the same time, such information can be crucial for tracking trends and reporting adverse health effects both on individual as on group or population level.

The overall aim is to influence policies, preventive measures and behaviours to promote best practices [13]. National and international guidelines aid the development of quality surveillance programmes, stimulate international harmonization and support the evaluation of health surveillance programmes in many tunnel projects worldwide. The International Tunnelling and Underground Space Association (ITA) has published several international guidelines for CAW that reflect the consensus in this field [13]. It is unclear, however, whether these guidelines have been implemented as recommended.

A scoping review was performed to systematically map and analyse studies and other information sources and to identify knowledge gaps. The aim was to investigate if publicly available reports and studies published in the past 50 years provide information on adverse health effects and occupational health surveillance related to CAW in caissons and during tunnel construction.

The following research questions were formulated:

Which adverse health effects are reported in publications on CAW in caissons and during tunnel construction? The availability of appropriate information about exposure parameters deserves special attention.

Which occupational health surveillance measures, activities or programmes are reported, and which results have been recorded?

## Methods

Scoping reviews and the presentation of their results were based on the guidelines described in the Joanna Briggs Institute Reviewers’ Manual [16]. Additionally, the components of this review were reported using the PRISMA Extension for Scoping Reviews Checklist [17]. In an iterative process, the entire research team drafted and revised a review protocol.

Information was obtained from studies of any design published in peer-reviewed scientific journals, and in the grey literature, including reports, guidelines, policy documents, overviews of tunnel construction projects and proceedings of international conferences.

To be included, publications must have reported on health surveillance and other preventive and coping strategies or on risks and adverse health effects of CAW during caisson and tunnel work. Publications published between 1974 and 2024 and written in English, German, French or Dutch were included. Abstracts without a full-text publication and animal studies were excluded.

A comprehensive systematic literature search was performed to screen scientific articles and grey literature published from 1974 to 2024. Relevant documents were identified using the following search engines, bibliographic databases and other resources: Ovid MEDLINE, Ovid Embase, Web of Science, PubMed/MEDLINE, Google, Ecosia, and websites of expert institutions and companies involved in CAW. A preliminary search strategy was developed with the assistance of an experienced research librarian (J.D.). To identify relevant key terms in titles and abstracts for the comprehensive search strategy, Ovid MEDLINE was subjected to a limited search to identify relevant articles. The final search strategy for Ovid MEDLINE, Ovid Embase and Web of Science is detailed in Table 4 (available as Supplementary data at *Occupational Medicine* Online). To explore additional publications, a second MEDLINE search was performed by one of the authors (F.v.D.) using an alternative focused search strategy in PubMed (Table 4, available as Supplementary data at *Occupational Medicine* Online). All results were exported and duplicates were removed. To identify further relevant materials, the search was supplemented by hand-searching key journals and scanning the reference lists of important publications.

Relevant studies were identified and selected using the web application Rayyan [18]. All authors screened the first 100 records to test feasibility and mutual agreement. Subsequently, the titles and abstracts of all records were independently screened for relevance by two reviewers (T.R.V. and P.J.v.O.) based on the previously described inclusion and exclusion criteria. For every excluded article, at least one reason for exclusion was noted. Any disagreements about the reasons for exclusion were discussed by the reviewers; if necessary, a third reviewer (C.H.) made the final decision. Full-text copies of all included studies were downloaded and independently screened for relevance by two reviewers (T.R.V. and P.J.v.O.). Disagreements were discussed among all researchers until consensus was reached. The reference lists of all included papers were screened for additional relevant studies by two reviewers (T.R.V. and P.J.v.O.). These reviewers collaboratively created a data-charting form to identify themes, charted the data independently, discussed results and refined the form. Data were extracted on (i) workplace typology characteristics (e.g. tunnel, caisson and pneumatic caisson), (ii) DCS and indicators of exposure (e.g. information on hyperbaric conditions), (iii) outcomes and (iv) characteristics of health surveillance measures.

## Results

**Figure 1.**
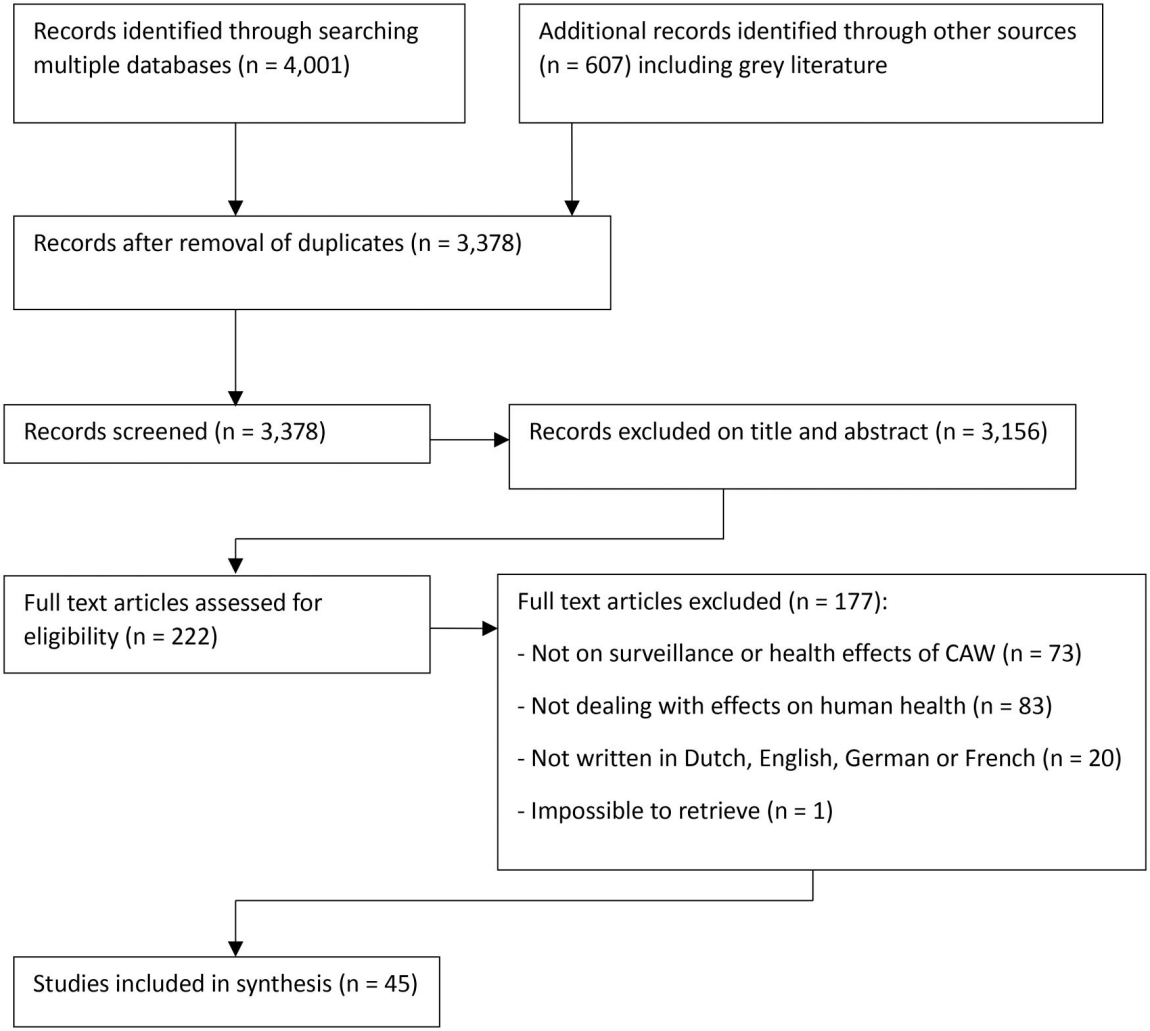
Flow diagram of study selection.

After removing duplicates, 3378 citations were identified from the searches, references and later findings, including grey literature. Based on the titles and abstracts, 222 full-text publications were selected for retrieval and evaluation of eligibility. Of these, 177 were excluded because they failed to meet at least one of the inclusion criteria. The remaining 45 studies were considered eligible for inclusion in this review (Figure 1).

Most studies conducted over the past 50 years concentrated on the registration, prevention and treatment of DCS, along with the occurrence and prevention of DON. Table 3 (available as Supplementary data at *Occupational Medicine* Online) shows studies containing quantitative information on adverse health effects related to CAW, preferably with appropriate information on exposure [1,4–8,19–43]. The adverse effects of CAW resulted primarily from changes in pressure. Several studies reported risks besides CAW, and a few described related health effects. Although activities related to the construction of underground projects had increased, only a limited number of projects were mentioned in the scientific literature [44]. Around 1975, the first TBMs came into use. Table 1 presents an overview of studies on TBM projects.

**Table 1.** Overview of studies on health and safety for workers in Tunnel Boring Machine Projects

Year of start construction	First author (year of publication) and reference number	Country	Project/Town	Max. pressure in bar (g)	Max. pressure in kPa absolute	Bounce and/or saturation	Man exposures	Deco table air, air/ox, Trimix, Heliox	Number of DCS	BIR = number of DCS/man exposures (%)
1975	Lam (1985) [6]	CN	Hong Kong tunnel	2.6	355	Bounce	232 661	Air	1187	0.5
1982	Kessel (1982) [45]	DE	Münchener U Bahn	1.5	250	Bounce	7225	Air	54	0.7
1982	Kessel (1986) [20]	DE	Münchener U Bahn	1.5	250	Bounce	41 000	Air	135	0.3
1982	How (1990) [21]	SG	Singapore tunnel	2.4	340	Bounce	188 538	Air	164	0.1
1984	Lo (1987) [24]	CN	Mass transit railway in Hong Kong.	5.0	600	Bounce	390 769	Air	2032	0.5
1985	Lam (1988) [6]	CN	Hong Kong tunnel	3.3	430	Bounce	154 390	Air	793	0.5
1990	Rϋegger (1994) [26]	CH	Grauholz tunnel Bern	3.4	440	Bounce	1961	Air	11	0.5
1990	Mathieu (1990) [53]	FR	Metro Lille	1.8	280	Bounce	3000	Air	0	0
1992	Andersen (2002) [30]	DK	Great Belt tunnel Sprogo	3.0	395	Bounce	1798	Air	7	0.4
1992	Andersen (2002) [30]	DK	Great Belt tunnel (deep part)	4.8	580	Bounce	7220	Air/ox	6	0.1
1996	Le Péchon (2010) [37]	RU	Metro tunnel line 1 St Petersburg	5.8	680	Bounce	118	23/53/24	4	3.4
1997	Faesecke (2003) [31]	DE	Elbe Rohre 4 tunnel Hamburg	4.5	550	Bounce	3367	Air/ox	21	0.6
1999	Bennett (2002) [29]	AU	Sydney tunnel	5.0	602	Bounce	3389	Air/ox	8	0.2
1999	Van Rees Vellinga (2006) [28]	NL	Western Scheldt tunnel	4.2	520	Bounce	1103	Air/ox	5	0.5
1999	Van Rees Vellinga (2006) [28]	NL	Western Scheldt tunnel	4.6–4.8	460–480	Bounce	52	25/50/25	0	0
1999	Van Rees Vellinga (2008) [35]	NL	Western Scheldt tunnel (excursions)	6.9	790	Saturation	402	12/48/40	2	0.5
1999	Van Rees Vellinga (2008) [35]	NL	Western Scheldt tunnel final deco	4.0	500	Saturation	44	9/73/18	0	0
2005	Le Péchon (2010) [37]	US	Brightwater Seattle	4.7	570	Bounce	Unknown	Air/ox	Unknown	Unknown
2005	Le Péchon (2010) [37]	US	Brightwater Seattle	5.5	650	Bounce	138	20/47/33	1	0.7
2008	Le Péchon (2010) [37]	US	Lake Mead Las Vegas	14	1500	Saturation	Unknown	He/O_2_	Unknown	Unknown
2011	Kulkarni (2017) [41]	IN	Chennai Tunnel India	1.2–2.0	220–300	Bounce	35 000	Air	3	0
2015	Imbert (2022) [43]	CN	Tuen Mun-Chek Lap Kok Hong Kong	3.5–5.7	450–670	Bounce/saturation	Unknown	20/47/33	0	0
2016	Mirasoglu (2018) [42]	TR	Eurasian tunnel Istanbul	4.0–7.3	500–830	Bounce	1007	6/77/17	0	0
2016	Mirasoglu (2018) [42]	TR	Eurasian tunnel Istanbul	7.3–10.8	830–1180	Bounce/saturation	117	4/84/12	0	0

deco table air, breathing air on decompression; deco table air/ox, breathing air alternated with oxygen breaks; trimix, gas mixture of nitrogen oxygen helium; heliox, gas mixture of helium and oxygen.

Decompression sickness is a major adverse health effect of CAW (Table 1). Caisson projects with compressed air, such as the Münchener U Bahn project (1982) [45], with a depth of 15 metres sea water (msw) (1.5 bar(g)) had a bends incidence rate (BIR) of 0.7%. In 1997 Milwaukee, USA 94 cases of DCS were reported, corresponding to a BIR of 1.4% (Table 1) [8]. Prior to 1974, compressed air was used as a breathing gas for caisson and TBM work (Table 1). Exposure to higher pressure has been noted in recent years for TBMs (Table 1). The results from the Chennai Tunnel project (2017) were noteworthy, despite compressed air (blackpool tables) being used, resulting in a BIR of 0% at a maximum depth of 19.5 msw. Kulkarni indicates that efficient supervision, lock operations by experienced operators and reduced working hours under pressure might have been the reasons for this low incidence. He also stated that ‘Employees often keep quiet because of fear of reprisal by the employer and occupational health is rarely addressed by the employers’ [41]. Tunnel and caisson work with compressed air could be performed at a maximum depth of 34 msw. As depth increased, the BIR reached 0.6% in other projects. BIRs were even higher during projects with workers at greater depths or with extended exposure times, such as the Great Belt Tunnel project in Denmark (1992).

Table 3 (available as Supplementary data at *Occupational Medicine* Online) shows that DON was a significant hazard in tunnel and caisson work [4]. For example, the prevalence of bone lesions in formerly exposed workers was reported to be 45% (56/123) in 1965 and 78% (96/123) in 1972 [5]. Divers with more than 5 years of experience were notably affected (54%), with the prevalence of DON being related to the diving depth [5]. A study in 1989 reported that the prevalence of DON was 18% among 2534 compressed air workers in tunnel construction and 4% among 4980 commercial (wet) divers [1]. Use of compressed air at pressures higher than 2.5 bar (g) was found to cause DON in tunnel workers [27]. Following the introduction of new air/oxygen decompression in 1986, the incidences of DON and DCS declined [7].

In the early years of CAW, compressed air was employed as breathing gas during decompression. Table 1 indicates that use of intermittent oxygen during decompression became standard practice in 1992. Work at greater depths necessitated the use of mixed gases as breathing gas during work under pressure to prevent nitrogen narcosis. This trend was evident in studies dating back to 1996. Most studies reported the use of Trimix, a breathing gas composed of oxygen, nitrogen and helium, although in varying proportions. To allow sufficient working time at great depths, saturation techniques were introduced in 1999. Workers are transported from the habitat to the bulkhead of the TBM by a mobile decompression chamber. The pressure differences between the habitat and workplace are relatively small. Therefore, the bottom time is easily sufficient at increased depth [35].

More conservative French (air) decompression tables were used in the deeper part (35 msw) of the Great Belt Tunnel project (1992), with a BIR of 0.1% [30]. The development of these decompression tables introduced the intermittent use of oxygen during decompression, resulting in a decreased BIR and shorter decompression times (Table 1). The Sydney Airport Tunnel project (1996), which involved work at a maximum depth of 50 msw, reported eight cases of DCS, with a BIR of 0.3% [29], with these low numbers being due to the use of oxygen during decompression.

Specific guidelines were developed by the ITA in 2024 for exposure to pressures higher than 3 or 4 bar (g) [13]. The introduction of mixed breathing gases, such as Trimix, and saturation techniques between 1996 and 2024 was designed to address the increased health and safety risks associated with exposure to higher pressures (Table 1). Trimix was used in the Westerscheldt Tunnelling project (Netherlands 1998) at pressures ranging from 4.6 to 4.8 bar (g) and during saturation excursions, with no cases of DCS observed [34,35]. Following the final decompression, however, after 2 or 3 weeks living in the habitat with excursions to the work site under high pressure, two cases of DCS were recorded (Table 1). Similar results were observed during the Eurasian Tunnel project (2014) and the Tuen Mun-Chek Lap Kok Link project (2015) in Hong Kong [42,43].

Another adverse health effect of CAW is barotrauma to the ears and sinuses [21,46]. Dental barotrauma is an adverse effect of CAW [40], and a study investigating the correlation between CO_2_ levels and DCS reported that high CO_2_ levels in the main lock during decompression were associated with 3-fold higher BIR [19]. Finally, CAW can impose significant stress on the ­cardiovascular system [24].

Pressure-unrelated risks and adverse health effects are considered new, emerging risks for compressed air workers. Modern tunnel construction is becoming increasingly mechanized, as seen with the shotcrete lining method to reinforce tunnel walls. Full-shift exposure to shotcrete can significantly increase airway resistance by 10% [45]. The silica content of bentonite can be as high as 30%, and exposure has been associated with higher risks of silicosis, chronic obstructive pulmonary disease and lung cancer [47]. A temporary myopic shift has been observed in caisson workers, similar to observations in patients treated with hyperbaric oxygen [48], with this myopia thought to be caused by increased pressure on the eye [49]. Infections pose a significant threat to the health and safety of compressed air workers in saturation. Identified bacteria include *Pseudomonas aeruginosa*, *Klebsiella*, *Staphylococcus aureus* and *Enterobacteria*, with some measurements detecting more than 100 Colony Forming Unit [38]. A stringent programme is necessary to identify and treat sources of infection [28,38,50].

The lack of published health surveillance programmes following the ITA guidelines is a problem. The guideline template for health surveillance programmes was partially followed in six studies [51].

Worldwide, to control costs, contractors have been outsourcing and subcontracting. In general, studies show that outsourcing of work activities has made working conditions more precarious, resulting in increased disease and accident rates [52]. None of the publications included in this survey analysed the effects of outsourcing on worker health during tunnelling projects. Many accidents were reported in a recent Dutch tunnelling project, resulting in a report of an official research committee of the Dutch province of South Holland [14]. This report stated that, because the project involved about 200 subcontractors, no one was responsible for a systemic process of risk management and control, including safety risks.

## Discussion

**Figure 2.**
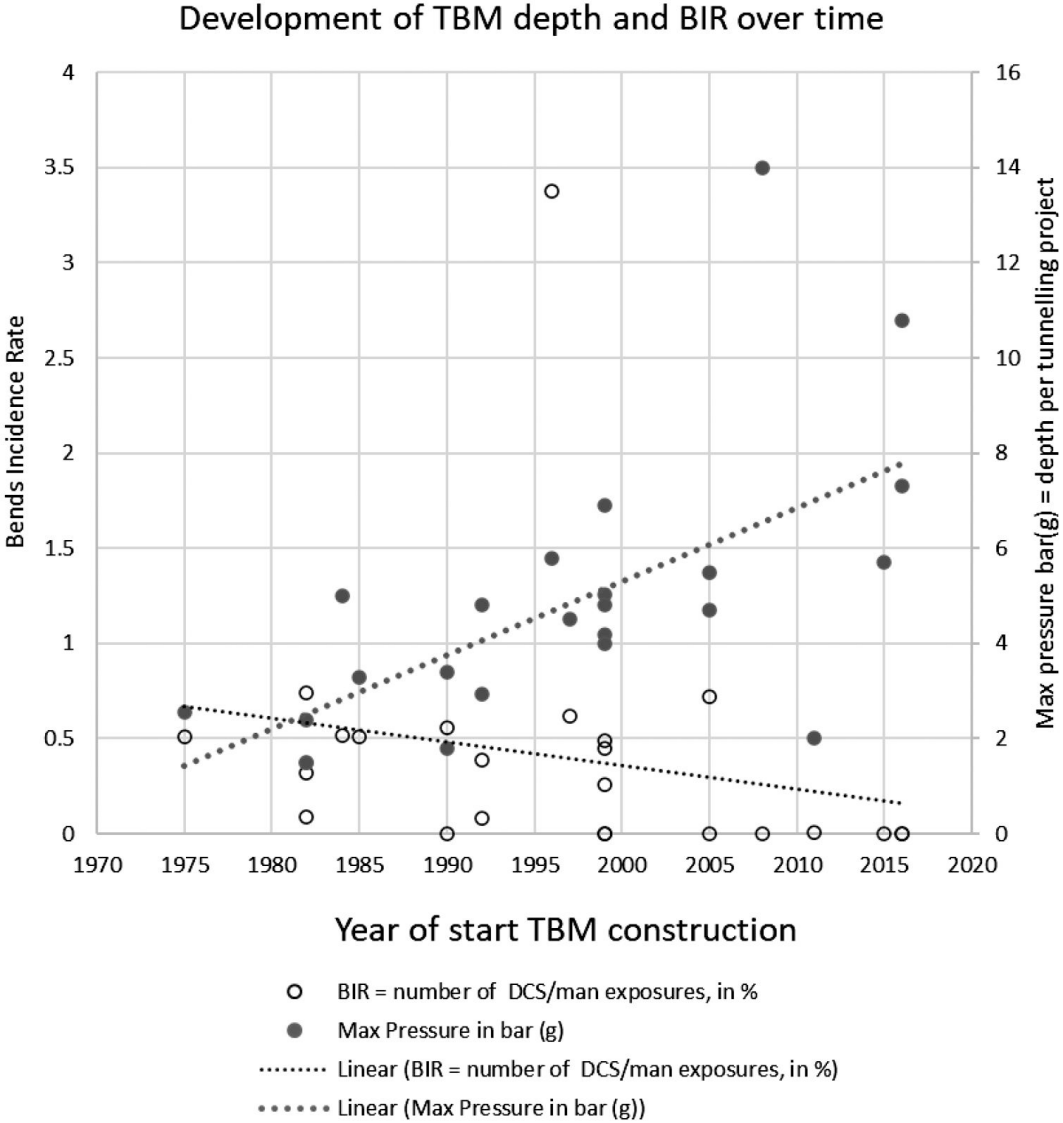
Numbers of compressed air tunnel work projects and incidence rates of the bends from 1970 to 2020. Twenty-four TBM projects were included. The black bullets represent the maximal pressure (depth) in a specific project, showing that the maximal depth increased markedly over time, from 108 to 140 msw. The white bullets represent the incidence rates of the bends, which showed an obvious downward trend, indicating that preventive measures and health surveillance resulted in markedly reduced adverse health effects.

This is the first review of publications on CAW in caissons and during tunnel construction covering the past 50 years. This review identified publications on risks, adverse health effects and the application of various health surveillance measures. Appropriate information about exposure parameters, including depth and exposure time, on DCS and DON rates were obtained from the scientific publications included in this review (Table 1, Figure 2). This information should be more extensive because of the large number of tunnels constructed in compressed air.

Occupational health surveillance programmes in CAW primarily focus on pressure-related health effects. Health surveillance measures were reported in 29 of the 45 included studies, although only 11 of these studies described a comprehensive health surveillance approach. The availability of comprehensive guidelines encourages the recording and reporting of project characteristics, expected risks, proposed preventive measures and the subsequent evaluation of efficacy and effectiveness. Several studies evaluated the use of more effective air/oxygen decompression tables, and other separate measures, such as the introduction of Trimix breathing gases, and the prevention of high CO_2_ levels during decompression [19].

The introduction of TBMs heralded a new era of mechanized tunnel construction. Tunnel boring has evolved from physically demanding labour to the management of sophisticated technological processes. Not only were lower-educated manual workers needed, but highly educated technicians were required to operate large, complex machines. Deep tunnel boring has led to working under increasingly higher pressures, particularly for maintenance and control tasks before the cutterhead, necessitating protective measures and continuous monitoring. In these new circumstances, compressed air workers are increasingly exposed to dust (shotcrete and bentonite), biological infections (Legionella and Pseudomonas), CO_2_ and/or heat stress and nitrogen narcosis which poses increasing safety risks [9,29,51,54].

The strength of this study lies in its systematic search and information extraction of peer-reviewed publications. Because the peer-reviewed literature does not include all relevant publications for a scoping review, the grey literature was also explored. The significance of this study includes its emphasis on the adverse pressure effects of compression on worker health, its importance for future health surveillance, including the effects of the professional qualities of the workers, optimal engineering and the development of a data registration system. Initial tunnel projects involving compressed air used air decompression tables, commonly known as Blackpool Tables. Over time, these tables were modified to incorporate longer decompression times and regulated ascent rates. Additionally, measures were implemented to ensure that workers exposed to compressed air maintained optimal fitness. Such measures included rigorous pre-employment examinations and precise monitoring of exposure levels [1,3–8,19,22,24,25,27,30,53]. Frequent pressure-related health effects of TBM and caisson work include barotrauma, dental barotrauma and temporary myopic shift [40,48]. The introduction of oxygen during decompression proved to be a revolutionary development. BIRs decreased significantly, shorter decompression times became feasible, and the long-term prevalence of DON declined [29,30,32,34–36]. The risks of DCS and DON continue to be a serious concern. Ongoing preventive efforts remain crucial, particularly given the increasing number of tunnels being constructed and the rising demand for deeper tunnel projects. Mixed gases have been employed to mitigate the risk of DCS at great depths, while avoiding the narcotic effects of nitrogen [32,34,35]. In a few projects, the saturation method from the offshore diving industry has been adapted to facilitate adequate working times at extreme depths.

The limitations of this study include the relatively small number of publications, as well as the potentially selective reporting of risks, adverse health effects and preventive measures in this field. Considering that ∼6700 tunnelling projects have been conducted worldwide from 1975 to the present, the number of scientific papers and reports is low [55]. Significant documents may have been overlooked. A language barrier impeded access to documents in languages such as Chinese, Spanish and Portuguese. The authors also lacked access to the registers and records of tunnelling companies and relevant authorities, including labour inspections. Furthermore, an international institution that collects data related to risks, health effects, accidents and preventive measures is currently lacking.

The quality of future studies merits attention. Because the present review did not systematically assess the quality of the articles, some of these may have been of low to moderate quality. This may limit the validity of the outcomes in some studies and may obscure possible selective reporting of risks, health effects and preventive measures in this field. More importantly, precise information about the exposure and characteristics of the study population was missing in some of the included studies. In before-and-after studies, the impact of physically demanding work was not evaluated.

The ITA Working Group No. 5 on Health and Safety in Works, in collaboration with the Compressed Air Working Group of the British Tunnelling Society, has developed guidelines for planning health and safety management for underground works and for promoting good working practices in highly compressed air environments [15,56]. High-pressure compressed air (HPCA) is a new field, in which work is performed under very high pressure (>4 bar (g)). The HPCA document recommends that, upon the completion of a project, the contractor should prepare a report assessing the effectiveness of the implemented decompression regimes. This report should be based on a statistical analysis of exposure data and post-decompression monitoring of workers. In daily practice, it remains unclear whether the ITA guidelines are being followed in different countries. Limited information is available about compliance with ITA guidelines. Employers generally underreport occupational injuries and diseases, possibly because of their limited understanding of guidelines or of legal requirements to maintain records on workplace injuries [54]. Underreporting of occupational diseases by physicians is also a problem, possibly because physicians must sign non-disclosure agreements prohibiting reporting [15]. Other reasons may include a lack of awareness of reporting guidelines, and the time and effort involved in reporting. Although level of education of occupational health physicians did not considerably increase reporting, a reminder on their legal obligation may be more effective [54].

Compressed air workers must be well trained to prepare for various pressure situations using practical familiarization programmes or protocols. The physical condition of compressed air workers is important because well-trained workers produce fewer bubbles during decompression and face a lower risk of DCS [22,39]. Comprehensive medical supervision can prevent work-related diseases [34]. To formulate new decompression tables, Doppler ultrasound examination is recommended to assess the bubble load following exposure. Intra-vascular bubbles interact strongly with sound waves. Nishi (DRDC Canada) has given comprehensive reviews of the history of ultrasonic methods for decompression surveillance [57]. This provides an opportunity to take appropriate measures in order to tailor decompression tables for specific working conditions [35,36].

Compressed air workers can experience adverse effects due to exposure to dust (shotcrete and bentonite), biological infectious agents (*Legionella* and *Pseudomonas*), CO_2_ or heat stress [9,28,50,58]. A clear occupational hygiene policy is needed to control exposure.

The risks associated with CAW can be minimized through optimal engineering. Examples include automatically changing the cutter head and inspecting the cutter wheel using cameras. When automated technical adjustments are exhausted, exposure of compressed air workers to increased pressure should be minimized [4,55].

Specific tunnel projects require customized decompression tables, applying compressed air (for caisson work) or gas mixtures like Trimix and Heliox (for deep TBM projects) [30,35]. Companies generally use proprietary decompression tables that are not publicly disclosed. These decompression tables may be made public once the project has concluded. These tables should also be evaluated by assessing the dependence of BIRs on air pressure, exposure time and other parameters. Exposure data and BIRs must also be reported to evaluate the measures taken. Custom-made decompression tables for new projects should be evaluated by Doppler ultrasonography, providing insight into the presence of bubbles in workers’ bloodstreams. The results of these assessments, including Doppler examinations, should be reported to the requisite national authority and to an international centre of expertise in CAW. This may result in adjustments to the diving tables’ algorithms, further reducing the risk of DCS [34–36].

On completion of all HPCA work valuable experience may be lost. ITA states as its mission to lead, advocate and facilitate the development of sustainable and innovative solutions …, enabling the tunnelling industry and its stakeholders to excel in delivering, (among others) …., ‘best practices in health-and-safety’. One of the tools is publication of guidelines, training, and creating platforms for exchanging information and ideas [56]. The ITA could propose the setting up of an international health and safety data registration system. The data collection should be organized in a standardized manner in all projects to allow data pooling, thereby providing insight into the circumstances and employees affected. A protocol is needed to protect workers’ privacy and legitimate specified commercial interests. At the end of the project, the reports about health and safety issues should be presented as peer reviewed publications and be accessible for independent research. A panel of experts under supervision of ITA could stimulate and coordinate the production of an annual report. Furthermore the system can build upon the work produced by the Compressed Air Working Group, which managed the Occupational Decompression Sickness Central Registry in the UK [10]. Another model is the registry system established in 1996 by the International Marine Contractors Association (IMCA), which collects anonymized incidents or near-misses in the diving and offshore industries [59]. The IMCA guidelines for health and safety also include reporting health surveillance information in the registration system. Finally, an international agreement, potentially supported by the International Labour Organisation, is needed to ensure adherence to high-quality guidelines for policy, practice and reporting, which may be included in future contracts.

International collaboration, coordinated by an independent international expertise centre on health and safety in CAW, working in partnership with universities, may be helpful. This type of international collaboration can draw from experiences with international cooperation in occupational and diving medicine. Well-designed and -managed international collaboration offers many advantages, including quality enhancement, cost reduction, scalability to deal with limited numbers of cases and prompt dissemination of results. Long-term health effects may be evaluated with cohort and case–control studies that assess exposure and health data of previously exposed workers. The ITA may play a key role in organizing a certified membership for tunnel boring companies as a quality mark for clients, governments, workers’ organizations, insurers and other stakeholders. The tripartite International Labour Organisation may also provide support. Governments and financiers must make adherence to the ITA guidelines a condition when awarding tunnel contracts. This will motivate tunnel builders and contractors to respect the ITA guidelines inclusive the reporting of data. This could be a pathway to get the international compressed air work community on the same page.

Table 2 presents a glossary of terms and abbreviations related to compressed air work and health risks.

**Table 2.** Glossary of terms and abbreviations related to compressed air work and health risks

Item	Abbreviation	Meaning
Bar	bar	Bar is a metric unit of pressure, defined as 100 000 Pa (100 kPa), though not part of the International System of Units (SI).
Bar gauge	bar (g)	Bar (g) = gauge pressure is defined as the difference between absolute pressure and atmospheric pressure. It measures how much higher or lower a gas pressure is than atmospheric conditions. In daily practice of compressed air work, bar (g) is the preferred pressure unit when referring to relative overpressure.
Bends		The term bends refers to injuries caused by a rapid decrease in pressure, causing pain and function loss in workers. Bends are part of the signs and symptoms of decompression sickness in workers.
Bends incidence rate	BIR	The (observed) incidence rate of decompression sickness over a specified period or in a specified project is the number of bends observed divided by the number of worker exposures (the number of times that one worker has been exposed to compressed air work in that specified period of the project).
Bounce dive		A bounce dive is one descent to some depth which remains, or is considered to be, essentially constant until the final ascent to the surface.
Caisson		A watertight chamber used in construction work under water or underground that is open at the bottom.
Caisson work		Construction work under water or underground in a caisson containing air under pressure to work in the dry.
Compressed air work	CAW	Work performed in compressed air conditions.
Decompression illness	DCI	All ill-health conditions resulting from exposure to compressed air or associated with the decompression process after exposure to high air pressure conditions.
Decompression sickness	DCS	Decompression sickness can originate during the decompression process, caused by gas previously dissolved in blood or tissues, forming bubbles in blood vessels during decompression. The main symptoms and signs are fatigue, weakness, joint and muscle pain, vertigo, but also include serious diseases such as hemiplegia.
Dysbaric osteo necrosis	DON	Dysbaric osteonecrosis is a type of avascular necrosis of the bone, most commonly found in undersea divers and workers after working in compressed air. The effect is not acute but appears months or years after exposure.
Habitat		A habitat in a saturation complex is a living chamber under pressure for compressed air workers.
High-pressure compressed air	HPCA	Working in an air pressure that is higher than usual in CAW work, in general higher than 3 or 4 bar (g)
Kilo Pascale (kPa)		kilopascal (kPa) as a unit of pressure measurement, k Pascal is a pressure of k newton per square metre in SI base units.
Metres sea water	msw	Depth in metres sea water
Saturation complex		Composition of pressure vessels, a shuttle and a pressurized work chamber
Saturation exposure	‘Sat’ exposure	A long duration exposure during which the exposed person is saturated with inert gases, lives at a storage pressure in the saturation habitat and can make transfers (excursions) under pressure to and from the working chamber in the head of the TBM.
Saturation run		Term for a period of living in the saturation habitat, often maximal 28 days, from the start of compression in the saturation habitat until the decompression in the process of leaving the saturation habitat cautiously returning to normal pressure.
Saturation excursion	excursion	Saturation excursion is the procedure by which compressed air workers are excursed from storage depth in the saturation habitat, to working depth, which may be below or above the storage depth.
Storage depth		The depth in terms of air pressure in bar (g) in the saturation habitat.
Storage pressure		The pressure to be maintained in the saturation habitat when workers are living there.
Trimix	Trimix	An oxygen, nitrogen, and helium breathing mixture. The convention is that a Trimix is described as oxygen/nitrogen/helium.
Tunnel-boring machine	TBM	Tunnel-boring machine, a large machine used for excavating tunnels through hard rock, wet or dry soil or sand, in a single operation (full-face drilling).
